# Relationships between Total, Free and Bioavailable Vitamin D and Vitamin D Binding Protein in Early Pregnancy with Neonatal Outcomes: A Retrospective Cohort Study

**DOI:** 10.3390/nu12092495

**Published:** 2020-08-19

**Authors:** Melinda Fernando, Thisara G. Coster, Stacey J. Ellery, Deborah de Guingand, Siew Lim, Cheryce L. Harrison, Helena J. Teede, Negar Naderpoor, Aya Mousa

**Affiliations:** 1 Monash Centre for Health Research and Implementation (MCHRI), School of Public Health and Preventive Medicine, Monash University, 43-51 Kanooka Grove, Melbourne, Clayton VIC 3168, Australia; melinda.fernando@monash.edu (M.F.); ranasinghe.thisara@gmail.com (T.G.C.); siew.lim1@monash.edu (S.L.); cheryce.harrison@monash.edu (C.L.H.); helena.teede@monash.edu (H.J.T.); negar.naderpoor@monash.edu (N.N.); 2 The Ritchie Centre, Hudson Institute of Medical Research and Department of Obstetrics and Gynaecology, Monash University, 27-31 Wright Street, Melbourne, Clayton VIC 3800, Australia; stacey.ellery@hudson.org.au (S.J.E.); deborah.deguingand@hudson.org.au (D.d.G.)

**Keywords:** vitamin D binding protein, free vitamin D, bioavailable vitamin D, pregnancy, neonatal outcomes, birth weight, jaundice

## Abstract

Maternal vitamin D deficiency has been associated with adverse neonatal outcomes, however, existing results are inconsistent. Current data focus on total 25-hydroxyvitamin D (25(OH)D) as the common measure of vitamin D status, while additional measures including vitamin D-binding protein (VDBP) and free and bioavailable metabolites have not been explored in relation to neonatal outcomes. We examined whether VDBP and total, free, and bioavailable vitamin D metabolites in early pregnancy are associated with subsequent neonatal outcomes. In this retrospective analysis of 304 women in early pregnancy (<20 weeks gestation), demographic and anthropometric data were collected and total 25(OH)D (chemiluminescent assay), VDBP (polyclonal enzyme-linked immunosorbent assay (ELISA)) and albumin (automated colorimetry) were measured in bio-banked samples. Free and bioavailable 25(OH)D were calculated using validated formulae. Neonatal outcomes were derived from a medical record database. Higher maternal total and free 25(OH)D concentrations were associated with higher neonatal birthweight (β = 5.05, *p* = 0.002 and β = 18.06, *p* = 0.02, respectively), including after adjustment for maternal covariates including age, body mass index (BMI) and ethnicity (all *p* ≤ 0.04). Higher total 25(OH)D and VDBP concentrations were associated with a lower likelihood of neonatal jaundice (odds ratio [OR] [95%CI] = 0.997 [0.994, 1.000], *p* = 0.04 and 0.98 [0.96, 0.99], *p* = 0.03, respectively), but these were attenuated after adjustment for the above maternal covariates (both *p* = 0.09). Our findings suggest a novel association between free 25(OH)D and neonatal birthweight. Total 25(OH)D concentrations were also associated with birthweight, and both total 25(OH)D and VDBP were associated with jaundice, but the latter were not significant after adjustment. These results suggest a potential link between these metabolites and neonatal outcomes; however, further large-scale prospective studies are warranted.

## 1. Introduction

Maternal metabolism undergoes substantial adaptations during pregnancy to facilitate an optimal intrauterine environment for the fetus [1]. These adaptations include unique changes in vitamin D homeostasis, intended to meet the increased requirements for fetal bone mineral accrual and enhance systemic and local maternal tolerance to paternal and fetal alloantigens [2]. Vitamin D regulates cellular differentiation and apoptosis and can protect skeletal fetal development while providing antimicrobial defense and immunotolerance at the maternofetal interface, that is the uterine decidua [3,4]. The uterus itself expresses the vitamin D receptor (VDR) and the uterine immune system is critical for successful implantation and a continued pregnancy [3]_._ Maternal 25-hydroxyvitamin D (25(OH)D) represents the main source of vitamin D for the fetus, with cord blood 25(OH)D concentrations being on average 25% lower than maternal concentrations [5,6]. Vitamin D deficiency in the mother can, therefore, lead to uteroplacental dysfunction, with negative effects on the fetus, including low birthweight, higher risk of preterm birth and an altered susceptibility for diseases such as neurodevelopmental disorders and cancers later in life [7,8]. However, existing study findings are highly divergent, owing to wide heterogeneity in study designs and populations, and controversies regarding the applied measure of vitamin D status.

Although total 25(OH)D is currently the most common measure of vitamin D status, recent studies have questioned whether this is the most appropriate means of determining vitamin D status in certain conditions such as pregnancy [9,10,11,12]. Pregnancy is characterised by high estrogen concentrations and, in turn, the production of vitamin D binding protein (VDBP) is increased, since steroid hormones stimulate hepatic synthesis of VDBP [13,14,15]. VDBP is the main carrier of vitamin D and changes in VDBP may alter the concentrations of, and relationships between, total and free 25(OH)D (the unbound fraction) [16]; these altered relationships have been shown in pregnant women [17]. Moreover, the ‘free hormone hypothesis’ suggests that only free steroid hormones are physiologically active as their lipophilic properties enable them to diffuse passively across cell membranes [18]. Although the placenta expresses megalin (enabling uptake of VDBP-bound vitamin D) and exhibits extra-renal 1α-hydroxylase activity, it remains unknown whether maternofetal transfer of vitamin D through placental cells occurs primarily via megalin-mediated endocytosis of VDBP-bound 25(OH)D compounds, by diffusion of the free hormone, or by both mechanisms [19].

To date, studies have not established whether the naturally occurring increases in VDBP during pregnancy may influence the bioavailability of vitamin D for the fetus, and subsequently impact on neonatal health. Kim et al. [14] reported that bioavailable 25(OH)D (free plus albumin-bound fraction) was lower in pregnant women compared to non-pregnant women, whereas another study [20] reported no differences in free 25(OH)D between pregnant and non-pregnant women. Moreover, Bouillon et al. [21] measured total and free 25(OH)D concentrations in 40 women at four timepoints during pregnancy and found reduced free 25(OH)D at all timepoints, however, no differences in total 25(OH)D levels compared with non-pregnant controls. In pregnant adolescents [22], VDBP was elevated and inversely associated with free 25(OH)D, but free 25(OH)D was not superior to total 25(OH)D as a predictor of parathyroid hormone, 1,25(OH)_2_D, 24,25(OH)_2_D, or calcium. Conversely, Tsuprykov et al. [11] found associations between free 25(OH)D and bone and lipid metabolism and kidney function in healthy Caucasian pregnant women, concluding that free 25(OH)D was a more precise determinant of vitamin D status during normal human pregnancy than total 25(OH)D. Importantly, these studies did not assess whether maternal free or bioavailable metabolite concentrations were associated with adverse neonatal outcomes. It has been posited that VDBP may exhibit a lower affinity for its metabolites during pregnancy as a compensatory mechanism to enable the level of free 25(OH)D to remain balanced, despite increases in VDBP [13]; however, this is yet to be confirmed.

Collectively, these findings suggest that total 25(OH)D, while relevant, may not be suitable as a single measure to adequately reflect functional vitamin D status in certain conditions, including in pregnancy [23]. To further clarify the relationship between maternal vitamin D status and neonatal outcomes, measurement of free and bioavailable metabolites should be considered, particularly given the elevated concentrations of VDBP in the pregnant state. Yet, with the exception of very few studies examining the influence of VDBP gene polymorphisms on birthweight [24], autism spectrum disorder [25], and type 1 diabetes [26], to our knowledge, no previous studies have examined free or bioavailable vitamin D or circulating VDBP concentrations in relation to neonatal outcomes.

Here, we aimed to assess maternal VDBP, total 25(OH)D concentrations, and calculated free and bioavailable 25(OH)D in a well-characterised multi-ethnic cohort of pregnant women to test the hypothesis that early pregnancy concentrations of these metabolites are associated with subsequent neonatal outcomes.

## 2. Materials and Methods

### 2.1. Study Design and Population

This study is a longitudinal retrospective cohort study of 304 pregnant women. Datasets and pre-collected bio-banked samples were available from two separate populations of pregnant women and these were combined and analysed for the purpose of this study. These two populations were recruited from the same medical setting in the same geographical location with similar methods and information for analysis, and thus were deemed sufficiently homogenous to be combined. All women were aged 18 to 40 years, English speaking and with a singleton pregnancy.

The first population was derived from the Healthy Lifestyles in Pregnancy (HLP) study cohort [27], which comprised 228 women initially recruited from Monash Health (three large tertiary hospitals in Melbourne, Australia). These women were recruited at early pregnancy (<15 weeks gestation) for a randomised controlled trial (RCT) that aimed to optimise gestational weight gain and prevent postpartum weight retention. The women were classified as high-risk for the development of gestational diabetes based on a validated risk prediction tool [28] and were overweight (body mass index [BMI] ≥ 25 kg/m^2^ or ≥23 kg/m^2^ for high-risk ethnicities) or obese (BMI ≥30 kg/m^2^). Women who were morbidly obese (BMI ≥ 45 kg/m^2^), had pre-existing diabetes (any type) or other chronic medical conditions precluding participation were excluded. In the initial RCT, 103 women were included in the control group (receiving standard care). Data and samples for 91 of these control group women were analysed in the present study.

The second population was derived from the Creatine and Pregnancy Outcomes (CPO) study, which aimed to characterise creatine homeostasis in pregnancy in a low-risk pregnancy group [29]. This cohort was also recruited in early pregnancy (<20 weeks gestation) from Monash Health, for a prospective observational study aimed at characterising creatine homeostasis in low-risk pregnancies [29]. There was no exclusion by BMI, but participants with significant medical or obstetric history or with primary models of care outside tertiary public hospital care (e.g., private, shared, or GP/midwife care) were excluded, as were women who used creatine supplements (since the original CPO study focused on creatine in pregnancy). The CPO low-risk cohort recruited 282 pregnant women, of whom 18 subsequently withdrew or were excluded, and 264 remained involved until study conclusion. Of these 264 participants, data and samples were available for 213 women and these were included in the present study.

Hence, with the combination of both cohorts, the present study included an eligible total sample size of 304 women.

### 2.2. Ethics

Informed written consent was provided by all participants and all data were anonymised for use in this study. The HLP high-risk pregnancy study was approved by the Monash Health Research Advisory and Ethics Committee (07216C) initially in 2008, and again in 2019 for the current retrospective study (19674). For the low-risk CPO pregnancy cohort, ethics approval was obtained in 2015 from Monash Health (14140B) and Monash University (7785), and again in 2019 for the current retrospective study (HREC/51952/MonH-2019-169657(v2)).

### 2.3. Data Collection

Data were collected at <20 weeks gestation for both cohorts and included measurement of height and weight as well as questionnaires to assess demographic characteristics such as age, ethnicity, parity, smoking status, and medical history. Birth and neonatal outcomes (including birthweight, large/small for gestational age, neonatal hypoglycaemia, jaundice, APGAR [appearance, pulse, grimace, activity and respiration] scores, sex, admission to neonatal intensive care or the special care nursery) and mechanisms of birth (normal vaginal delivery, C-section, emergency C-section, induction of labour, instrumental) were retrieved retrospectively from the Birthing Outcomes System (BOS) database at Monash Health. Preterm births (<37 weeks gestation) were defined based on the Royal Australia New Zealand College of Obstetricians and Gynaecologists guidelines [30]. Large and small for gestational age neonates were those with weights > 90th and <10th percentile for gestational age, respectively [31]. All other outcomes were determined by routine clinical assessments as reported in the BOS database, which uses a standardized method of reporting perinatal data in Victoria, Australia [28]. Data were missing for some variables due to incorrect or missing entries in questionnaires or medical records or insufficient sample volumes and/or inaccurate assay results derived from the bio-banked samples.

### 2.4. Biochemical Analyses

All biochemical analyses were conducted in samples which had been collected at <20 weeks gestation and stored at −80 °C in departmental bio-banks. Total 25(OH)D concentrations were measured in serum samples by Monash Health Pathology using direct competitive chemiluminescent immunoassays on a LIAISON analyser (DiaSorin In., Stillwater, MN, USA) with external quality control based on Royal College of Pathologists of Australasia (RCPA) standards. Inter- and intra-assay coefficients of variation (CVs) were <10% and <4%, respectively.

Analysis of VDBP was conducted using polyclonal competitive enzyme-linked immunosorbent assays (ELISA, Abcam ab108853), according to manufacturer’s instructions. Briefly, samples were run in duplicate at random across nine plates. A sample pooled from six participants was also run on each plate to serve as an internal control (calibrator), used to determine the inter-assay variability and normalise measures across plates. Optical density readings were acquired for each standard and sample, by measuring an absorbance wavelength of 450 nm, immediately followed by a measurement at 570 nm to correct optical imperfections (SpectraMax i3, Molecular Devices). VDBP concentrations were determined based on a standard curve generated for each plate. Before correction, the intra- and inter-assay CVs were <12% and <22%, respectively. To account for inter-plate variability, all data were adjusted using an adjustment factor, in accordance with Zwirner et al. [32]. The adjusted results were used for all subsequent statistical analyses.

Albumin was analysed by Monash Health Pathology using an automated colorimetric method carried out on a Beckman Coulter AU5812 System, for the purpose of calculating free and bioavailable vitamin D values using the formula by Bikle et al. [33].

### 2.5. Calculation of Free and Bioavailable 25(OH)D

We calculated free 25(OH)D based on the formula by Bikle et al. [33] with the affinity binding constants for 25(OH)D with albumin and VDBP (6 × 10^5^ M^−1^ and 7 × 10^8^ M^−1^, respectively), determined using centrifugal ultrafiltration dialysis. The calculations used for free and bioavailable vitamin D and the relevant unit conversions are summarised below:

(1) $$VD_{free}=\frac{VD_{total}}{\left(1+\left(6\;\times 10^{5}\right)\times Alb\right)+\left(\left(7\;\times 10^{8}\right)\times VDBP\right)}$$

(2) $$VD_{bio}=\;\left(1+\left(6\;\times 10^{5}\right)\times Alb\right)\;\times \;VD_{free}$$

where:

$VD_{free\;}\;$= serum free 25(OH)D concentrations in mol/L;

$VD_{total}\;$= serum total 25(OH)D concentration in mol/L;

$VD_{bio}$ = serum bioavailable 25(OH)D concentration in mol/L;

Alb = serum albumin concentration in mol/L (albumin was measured in g/L and converted to mol/L using: g/L÷66430);

VDBP = serum vitamin D binding protein concentration in mol/L (VDBP was measured in ug/mL and converted to mol/L using: $\mathrm{ug}/\mathrm{mL}÷5.8\times 10^{7}$).

Free vitamin D is reported in pg/mL and was converted from mol/L to pg/mL using: $\mathrm{mol}/L\times 0.4166\times 10^{12}$. Total and bioavailable 25(OH)D are reported in nmol/L and were converted from mol/L to nmol/L using:$\;\mathrm{mol}/L\times 10^{9}$.

### 2.6. Statistical Analyses

Participant demographic, clinical and biochemical parameters including vitamin D metabolites are presented as mean ± standard deviation (SD) or frequencies (%). Shapiro–Wilk tests and visual inspection of histograms were used to assess normality and non-normal variables including total, free, and bioavailable 25(OH)D were logarithmically transformed to the base-10 to meet the assumptions of parametric testing prior to analysis.

Univariable associations between the continuous vitamin D metabolite concentrations and continuous (e.g., birthweight) or categorical (e.g., with or without jaundice) neonatal outcomes were analysed using general linear or simple logistic regression, respectively. Differences in mean concentrations of the vitamin D metabolites between binary outcome groups (e.g., women whose neonates had or did not have jaundice) were assessed using independent student’s *t*-tests. Variables which were significant on univariable analysis were further assessed in multiple linear and logistic regression models, with adjustment for predetermined maternal characteristics considered to be clinically relevant to the outcomes, including maternal age, BMI, and ethnicity. All statistical analyses were performed using Stata V.15.0 (Stata, College Station, TX, USA) and a two-tailed *p* < 0.05 was considered statistically significant.

## 3. Results

### 3.1. Sample Characteristics

Participant characteristics for the 304 women included in this study are presented in Table 1. Despite combining cohorts from two studies, there were no differences between the women from the HLP and CPO cohorts with respect to demographic characteristics including maternal age, BMI, parity, and ethnicity or any of the outcomes measured (all *p* > 0.05).

Participants had a mean maternal age of 31.4 ± 4.2 years (mean ± SD) and 46% were primiparous. Approximately 43% were of non-Caucasian ethnicity and 28.3% were classified as overweight, with 25.6% classified as obese. The mean serum total 25(OH)D concentration at early pregnancy was 54.8 ± 20.2 nmol/L. As per US Endocrine Society guidelines [34], 14.5% of participants had sufficient vitamin D status (total 25(OH)D ≥ 75 nmol/L), 46.8% were of insufficient status (50–74.9 nmol/L), 30.3% were deficient (25–49.9 nmol/L), and 8.4% were severely deficient (<25 nmol/L). Neonatal outcomes are summarised in Table 2, whereby 9.6% and 8.6% of neonates were small and large for gestational age, respectively, and 11.4% had jaundice.

### 3.2. Univariable Associations between Vitamin D Metabolites and Demographic Variables

Higher maternal age correlated with lower VDBP (r = −0.24, *p* < 0.01) and higher free and bioavailable 25(OH)D concentrations (r = 0.22, *p* < 0.01 and r = 0.24, *p* < 0.01, respectively). A higher BMI was associated with lower VDBP (r = −0.12, *p* = 0.04) and total 25(OH)D (r = −0.26, *p* < 0.001) but was not associated with free or bioavailable 25(OH)D (both *p* > 0.05). There were no associations between vitamin D metabolites and any other demographic variables (all *p* > 0.05).

Correlations between the vitamin D metabolites were also observed, whereby VDBP was inversely associated with free and bioavailable 25(OH)D (both *r* = −0.6, *p* < 0.001), but not with total 25(OH)D (*p* = 0.8). Total 25(OH)D concentration was positively correlated with free and bioavailable 25(OH)D (both *r* = 0.7, *p* < 0.001) and there was a positive correlation between free and bioavailable 25(OH)D (*r* = 0.9, *p* < 0.001).

### 3.3. Univariable Associations between Vitamin D Metabolites and Neonatal Outcomes

As shown in Table 3, higher maternal VDBP concentrations were associated with a lower likelihood of neonatal jaundice, whereby women whose neonates were born with jaundice had a lower mean VDBP concentration of 325.11 ± 119.56 µg/mL compared with 372.54 ± 126.08 µg/mL in women whose neonates did not have jaundice at birth (*p* = 0.04). There were no associations between VDBP and any other neonatal outcomes (Table 3).

Total 25(OH)D was positively correlated with higher birthweight (*p* = 0.002) and was also inversely correlated with jaundice, whereby women with neonates with jaundice had a lower mean total 25(OH)D of 47.51 ± 18.15 nmol/L compared with 55.75 ± 20.4 nmol/L in women whose neonates did not have jaundice (*p* = 0.03).

Higher free 25(OH)D was also associated with higher birthweight (*p* = 0.02), but not with any other neonatal outcomes including jaundice (Table 3). Bioavailable 25(OH)D was not significantly associated with any of the recorded outcomes, although a trend was seen with birthweight (*p* = 0.08; Table 3).

### 3.4. Multivariable Analyses of Vitamin D Metabolites and Neonatal Outcomes

Results of multivariable logistic and linear regression analyses for neonatal outcomes of interest are shown in Table 4. These models were adjusted for predetermined confounders based on clinical relevance to the pregnancy outcomes, including maternal age, BMI, and ethnicity.

Despite a univariable association between VDBP and jaundice, this relationship was no longer significant after adjustment for any of the covariates (*p* > 0.05 in all models, Table 4). The initial relationship between total 25(OH)D and jaundice remained significant after adjusting for maternal age (*p* = 0.02) but was attenuated after adjustment for BMI (*p* = 0.05) and ethnicity (*p* = 0.09; Table 4), as well as after additional exploratory adjustment for length of gestation (*p* = 0.2).

Total and free 25(OH)D remained associated with birthweight in the fully adjusted models accounting for maternal age, BMI, and ethnicity (Table 4) as well as after exploratory adjustment for length of gestation (*p* = 0.04 and *p* = 0.01, respectively). Bioavailable 25(OH)D was not associated with any neonatal outcomes in the fully adjusted models (Table 4).

## 4. Discussion

In the present study, we found that higher total and free 25(OH)D were associated with higher neonatal birthweight, the latter of which has not previously been reported. These associations remained significant after adjustment for maternal age, BMI, and ethnicity, highlighting the strength of the relationships. This was also the first study to investigate and identify associations between VDBP and jaundice. However, as this was attenuated by adjustment for maternal covariates, it is likely that underlying factors such as maternal age or BMI may be driving these relationships.

We identified a novel relationship between birthweight and the biologically functional vitamin D component, free 25(OH)D. We also report a positive association between total 25(OH)D and birthweight, which is consistent with some studies showing that vitamin D deficiency in early pregnancy was associated with decreased birthweight [35,36,37,38], although others report no relationship [39,40,41]. Similarly, some clinical trials have shown improvements in birthweight and growth indices in women receiving vitamin D supplementation [42,43], while others have not [44,45]. Importantly, none of these observational studies or clinical trials have differentiated between total and free 25(OH)D in their analyses, which may in part explain some of the inconsistencies in the evidence. Based on the free hormone hypothesis mentioned earlier, free 25(OH)D may influence birthweight directly via the VDR, since the VDR is a critical regulator of placental hormone secretion and uterine immunomodulation [46]. In normal placentae, the VDR is localised mainly in trophoblasts [47]. Through the VDR, free 25(OH)D may directly regulate trophoblast differentiation and human chorionic gonadotrophin (hCG) production as well as stimulating the secretion of other hormones including estradiol, progesterone and placental lactogen. These hormones support uteroplacental blood flow and placental neovascularization, thereby facilitating fetal growth [38]. Vitamin D also influences the production and secretion of insulin, which is a growth-promoting hormone during fetal life, with direct and indirect effects on adipogenesis and stimulation of insulin-like growth factors and their binding proteins, respectively [48,49,50]. Indirectly, vitamin D promotes maternal calcium absorption and placental calcium transfer (alongside other nutrients) to the neonate for muscle development and mineralisation of the fetal skeleton [38]. Together, these pathways suggest that inadequate concentrations of maternal 25(OH)D, particularly the free 25(OH)D component, may impair these actions or limit the beneficial effects of vitamin D in regulating uteroplacental function and fetal growth. That said, the impact of these metabolites on uterine and placental vitamin D metabolic homeostasis and subsequent fetal growth remains poorly understood. Moreover, given that total and free 25(OH)D were correlated and both were associated with birthweight, we could not distinguish if one measure was superior to the other in relation to this outcome. Further studies are warranted to clarify the relationship between vitamin D metabolites and fetal growth and to delineate the underlying cellular and molecular mechanisms.

We found no relationship between VDBP concentrations and neonatal birthweight, which is likely due to our analysis being based on circulating VDBP levels and not allelic variants of the *GC* gene, which have previously been linked with birthweight [24]. Some studies suggest that VDBP may influence birthweight via its role in placental dysfunction, since fetal growth restriction (FGR) is known to be associated with placental dysfunction, particularly in the later stages of pregnancy which coincide with a rise in VDBP [51,52]. Indeed, the single previous study examining VDBP and FGR reported significantly lower placental VDBP levels in pregnancies complicated with FGR [53]. It is possible that a lack of VDBP, particularly the absence of its usual peaks at the time of increased demand, may play a role in the development of placental dysfunction resulting in FGR [53]; however, this could not be confirmed with the present data. Further investigation, with larger samples and a greater range of birthweights as well as analysis of genetic variations and both serum and placental VDBP levels, may help to clarify whether there are any potential links between VDBP and birthweight.

Finally, we found a reduced likelihood of jaundice in neonates born to women with higher concentrations of total 25(OH)D and VDBP. One previous study has reported a relationship between vitamin D deficiency and jaundice [54], however, associations with VDBP have not been explored. It should be noted that the relationships between total 25(OH)D and VDBP with jaundice were attenuated by adjustment for maternal covariates, suggesting that factors such as BMI or ethnicity may be driving these relationships. Indeed, neonatal jaundice is more common in women with a higher BMI compared to those with a low BMI [55]. It is also possible that associations between VDBP and jaundice may reflect underlying prematurity since jaundice occurs more commonly in preterm infants [56], and VDBP has been associated with preterm labour [57,58,59] (though not significantly associated in this study). To consider this potential confounder, we adjusted for length of gestation or preterm labour in an exploratory model and this attenuated the association between both total 25(OH)D and VDBP with jaundice, highlighting that this may be the primary driving factor for the observed associations. Nevertheless, given the lack of available evidence, further studies analysing VDBP in relation to jaundice, with consideration of demographic factors and length of gestation, may help confirm this potential relationship, or lack thereof.

### Strengths and Limitations

Limitations of the present study should be acknowledged. The observational design and secondary post-hoc analysis of existing data precludes assessment of causality or additional collection of data or samples (including missing data for some variables), and no formal power calculation was performed. Despite combining two cohorts, the sample size is modest and may have been underpowered to detect associations between VDBP or total, free or bioavailable 25(OH)D with some of the neonatal outcomes analysed, especially for variables or outcomes with missing data. We could not assess temporal changes in vitamin D due to seasonality, sun exposure, supplement use, or diet during pregnancy since the study utilised a single measurement of vitamin D metabolites in early pregnancy (<20 weeks gestation). The exact gestational age at which vitamin D metabolites were assessed was not recorded and may influence the relationships observed [60], although we expect this to be minimal since all samples were collected at <20 weeks gestation. Genetic assessments were not conducted, thus, we could not characterise the potential contributions of vitamin D gene axis polymorphisms including in the VDR or *GC* genes. Although the CV values for the VDBP ELISA were likely too high for the results to be considered diagnostic, the randomisation of samples across plates and the use of calibrators allowed for accurate comparisons to be made between groups for the purpose of this hypothesis-generating study. Free 25(OH)D concentrations were calculated rather than directly measured and these calculations depend heavily on the measured VDBP. However, calculated free 25(OH)D determined by polyclonal VDBP assays (as used in this study) has been shown to correlate strongly with directly measured free 25(OH)D [61]. Total 25(OH)D concentrations were assessed using Diasorin assays rather than the gold-standard liquid chromatography method. Results should be interpreted in light of these limitations and further research utilising gold-standard methods including the newly developed ELISA for free 25(OH)D would be beneficial to validate these findings.

Despite these limitations, our study is the first to report a relationship between maternal free 25(OH)D and neonatal birthweight. The sample comprised a well-characterized cohort of women with various baseline risk profiles, which allowed examination of a diverse group of pregnant women. The multi-ethnic cohort reflects the cultural and linguistic diversity of the wider Australian population [62], which is important in vitamin D research since earlier studies in pregnancy were conducted mostly in Caucasian cohorts, despite knowledge of the interplay between ethnicity, genetics and the vitamin D metabolic system [63]. We were able to assess longitudinal relationships between vitamin D metabolites in early pregnancy and neonatal outcomes and we adjusted for potential confounding by maternal age, BMI, and ethnicity which have seldom been considered in previous studies. We used polyclonal assays which are considered more reliable in measuring VDBP than the more commonly used monoclonal assays, particularly in ethnically diverse cohorts, as they are less influenced by VDBP genotype polymorphisms [61].

## 5. Conclusions

In summary, the present study suggests a novel association between maternal free 25(OH)D concentrations in early pregnancy and neonatal birthweight. These findings are considered hypothesis-generating rather than confirmatory and, given that total and free 25(OH)D were correlated and both were associated with neonatal birthweight, our findings require validation in larger prospective studies to clarify whether one or a combination of these metabolites offers greater potential utility in predicting adverse neonatal outcomes. Future studies should incorporate large and diverse multi-ethnic cohorts with sufficient statistical power and frequent sampling using appropriate assays to trace metabolite concentrations throughout pregnancy and examine their impact on neonatal outcomes.

## Figures and Tables

**Table 1:** Sample demographic, anthropometric, and biochemical characteristics.

Variable	*n*	Mean ± SD or n (%)
Maternal age (years)	301	31.4 ± 4.2
**Parity**	289	
Primiparous		133 (46.0)
2		106 (36.7)
3		39 (13.5)
4		11 (3.8)
**Ethnicity**	**303**	
Caucasian		174 (57.4)
South East and North East Asian		37 (12.2)
Southern and Central Asian		69 (22.8)
Other ^1^		23 (7.6)
Past history of GDM	304	13 (4.3)
Smoker	304	3 (1)
BMI (kg/m^2^)	293	26.8 ± 5.9
Gestational weight gain (kg, at 28 weeks)	255	7.4 ± 3.6
**Vitamin D metabolites**		
Total 25(OH)D (nmol/L)	297	54.8 ± 20.2
Free 25(OH)D (pg/mL)	291	5.6 ± 4.7
Bioavailable 25(OH)D (nmol/L)	291	4.4 ± 3.1
VDBP (µg/mL)	298	364.7 ± 126.1
Albumin (g/L)	302	36.9 ± 4.1

Data reported as mean ± standard deviation or frequency n (%); ^1^ Other represents African, Middle-Eastern, European, South American and Polynesian. Sample sizes differ due to missing data (incorrect or missing entries) in the original studies or insufficient sample volumes or inaccurate assay results derived from the bio-banked samples. Abbreviations: GDM, gestational diabetes mellitus; BMI, body mass index; 25(OH)D, 25-hydroxyvitamin D; VDBP, vitamin D binding protein.

**Table 2:** Summary of neonatal outcomes.

Variable	Mean ± SD or n (%)
Gestation at delivery (weeks)	39.1 ± 2.0
Preterm birth	16 (5.4)
Birth trauma	4 (1.3)
**Mode of birth**	
Normal vaginal birth	161 (54.8)
Instrumental birth	58 (19.7)
Caesarean section	75 (25.5)
**Neonatal anthropometry**	
Large for gestational age (>90%)	28 (9.6)
Small for gestational age (<10%)	25 (8.6)
Very small for gestational age (<3%)	7 (2.4)
Head circumference (cm)	34.6 ± 2.1
Birthweight (g)	3360.6 ± 554.0
**Neonatal sex**	
Male	154 (52.6)
Female	139 (47.4)
**APGARs**	
Apgar 1 score (1 min after birth)	8.3 ± 1.5
Apgar 5 score (5 min after birth)	8.8 ± 1.0
Shoulder dystocia	8 (2.7)
Jaundice	34 (11.4)
Neonatal hypoglycaemia	10 (3.4)
SCN/NICU admissions	51 (17.3)

Data reported as mean ± standard deviation or frequency n (%) unless otherwise specified. Abbreviations: APGAR, Appearance, Pulse, Grimace, Activity, and Respiration; SCN/NICU: Special care nursery/Neonatal intensive care unit.

**Table 3:** Univariable associations between vitamin D metabolites and neonatal outcomes.

Variable	VDBP		Total 25(OH)D		Free 25(OH)D		Bioavailable 25(OH)D	
	β or OR (95%CI)	*p*	β or OR (95%CI)	*p* *	β or OR (95%CI)	*p* *	β or OR (95%CI)	*p* *
Birthweight	0.11 (−0.40, 0.62)	0.7	5.05 (1.90, 8.19)	**0.002**	18.06 (4.50, 31.6)	**0.02**	24.78 (4.10, 45.45)	0.08
Jaundice	0.997 (0.994, 1.000)	**0.04**	0.98 (0.96, 0.99)	**0.03**	0.99 (0.91, 1.10)	0.8	1.00 (0.89, 1.13)	0.9
Head Circumference	−0.001 (−0.003, 0.001)	0.4	0.008 (−0.006, 0.02)	0.2	0.05 (−0.004, 0.10)	0.09	0.08 (−0.009, 0.16)	0.1
SGA (<10%)	1.00 (0.99, 1.00)	0.8	0.99 (0.97, 1.02)	0.5	0.97 (0.85, 1.10)	0.6	0. 0.97 (0.82, 1.14)	0.7
LGA (>90%)	1.00 (0.99, 1.00)	0.9	1.01 (0.99, 1.03)	0.4	1.02 (0.96, 1.10)	0.5	1.05 (0.95, 1.16)	0.5
Neonatal hypoglycaemia	1.00 (0.99, 1.00)	0.5	1.01 (0.98, 1.04)	0.9	0.95 (0.77, 1.18)	0.6	0.95 (0.72, 1.24)	0.7

Data were analysed using general linear or simple logistic regression models for continuous and binary outcomes, respectively, and results are reported as beta coefficients or odds ratios with 95% confidence intervals and corresponding *p*-values; * *p*-values represent significance of analyses after vitamin D metabolite data were logarithmically transformed to the base 10 to approximate normality. Bold numbers denote statistical significance at *p* < 0.05. Abbreviations: VDBP, vitamin D binding protein; 25(OH)D, 25-hydroxyvitamin D; SGA/LGA, small/large for gestational age.

**Table 4:** Multivariable regression analyses of relationships between vitamin D metabolites and pregnancy outcomes after adjustment for maternal covariates.

Dependent Variable	Model	VDBP				Total 25(OH)D				Free 25(OH)D				Bioavailable 25(OH)D			
		β	SE	R^2^	*p*	β	SE	R^2^	*p*	β	SE	R^2^	*p*	β	SE	R^2^	*p*
Birthweight	+ age	0.10	0.27	0.001	0.7	5.15	1.60	0.03	**0.003**	18.80	6.97	0.03	**0.01**	26.68	10.74	0.02	0.06
	+ BMI	0.12	0.27	0.01	0.7	7.01	1.74	0.07	**<0.001**	20.72	7.05	0.04	**0.004**	22.28	10.88	0.04	**0.02**
	+ ethnicity	0.15	0.26	0.07	0.6	5.36	1.78	0.10	**0.004**	16.96	6.95	0.09	**0.04**	22.02	10.81	0.08	0.1
Jaundice	+ age	−0.003	0.002	0.02	0.07	−0.02	0.01	0.03	**0.02**	−0.02	0.05	0.01	0.5	−0.02	0.07	0.01	0.6
	+ BMI	−0.003	0.002	0.03	0.1	−0.02	0.01	0.04	0.05	−0.01	0.05	0.03	0.6	−0.01	0.07	0.03	0.7
	+ ethnicity	−0.003	0.002	0.04	0.09	−0.02	0.01	0.05	0.09	−0.001	0.05	0.03	0.9	0.004	0.06	0.03	0.9

Data are presented as unstandardized beta coefficients (β) with corresponding standard error (SE), and R^2^ (or pseudo R^2^ for logistic regression of binary outcomes). Plus (+) signs indicate addition of each variable to the model (e.g., for birthweight, the first row is a model adjusted for maternal age only, the second row is adjusted for maternal age and BMI, and so on). Bold numbers denote statistical significance at *p* < 0.05. Abbreviations: VDBP, vitamin D binding protein; 25(OH)D, 25-hydroxyvitamin D.
